# Resveratrol exerts no effect on inflammatory response and delayed onset muscle soreness after a marathon in male athletes. A randomised, double-blind, placebo-controlled pilot feasibility study

**Published:** 2014-04-08

**Authors:** M W Laupheimer, M Perry, S Benton, P Malliaras, N Maffulli

**Affiliations:** 1 Centre for Sports and Exercise Medicine, Queen Mary University of London, Barts and The London School of Medicine and Dentistry, Mile End Hospital, 275 Bancroft Road, London E1 4DG, England; 2 BUPA Center for Excellence Muskuloskeletal&Sports Medicine, London Basinghall Street, 4 Basinghall Street, London, EC2V 5BQ; 3 National Clinical Guideline Centre, Royal College of Physicians, London; 4 Barts Health NHS Trust, Department Clinical Biochemistry, Pathology and Pharmacy Building, Royal London Hospital, E1 2ES

**Keywords:** Resveratrol, Marathon, Immune Response, DOMS

## Abstract

**Objective:**

We investigated whether the inflammatory response and delayed onset of muscle soreness after a marathon are altered by resveratrol, a natural polyphenolic flavonoid antioxidant.

**Design:**

Double blind placebo-controlled randomised pilot study.

**Setting:**

London Marathon.

**Participants:**

Marathon race participants

**Interventions:**

7 healthy male athletes were randomised to receive Resveratrol (600 mg Resveratrol daily for 7 days immediately before the marathon) or a placebo.

**Main Outcome Measurements:**

Blood samples taken 48 hours before and 18–32 hours after the marathon were analysed for white blood cell count (WBC) and C-reactive protein (CRP). A VAS score was taken at the same times as the blood samples to assess delayed onset muscle soreness.

**Results:**

There were no significant differences between the two groups in terms of changes occurring between pre- and post- tests for WBC, CRP or VAS.

**Conclusions:**

There were no differences in immune response or delayed onset muscle soreness between resveratrol and placebo after a marathon. Further investigations are needed with longer treatment time and higher doses, analysing additional parameters such interleukins for a possible effect of resveratrol on the inflammatory response due to extensive exercise. To avoid a type II error, 17 subjects in each group would be required.

## INTRODUCTION

Running a marathon causes changes in immune response and microscopic damage to the muscles [1]. After prolonged running, substances released from injured muscle cells initiate an inflammatory response [2,3,4]. Neutrophils, macrophages and lymphocytes exhibit the greatest changes in response to marathon competition [2,3]. Changes in venous blood for up to 36 hours after the marathon show neutrophilia, lymphopenia and an increase in pro-inflammatory cytokines (e.g. IL-6) [2,3,4,5]. A study of 70 male and 20 female runners showed an increase in the white blood cell count (+160%) and C reactive protein (CRP) (+2000%) immediately and 24 hours after a 42 km marathon race [6]. Several other studies showed a marked increase of CRP 24 hours after a marathon [5,7].

Resveratrol is a food supplement found in the seeds and skins of grapes, red wine, mulberries, peanuts and rhubarb. It may provide numerous health benefits, including prevention of a variety of illnesses, including cancer, cardiovascular disease and ischaemic injuries [8,9,10]. Resveratrol showed anti-inflammatory and immune-modulating actions via effective inhibition of cyclo-oxygenase (COX) activity [8,9,10,11,12,13,14]. The anti-inflammatory activities of resveratrol are related to impaired neutrophil function, absence of up-regulation of tumour necrosis factor alpha (TNF-alpha) and reduced over expression of COX-2 [12]. These anti-inflammatory properties have to our knowledge not been evaluated in sports medicine.

**Figure 1:**
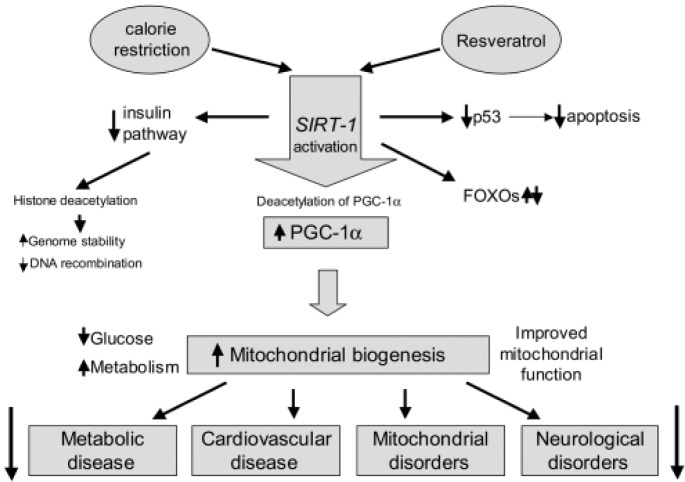
*SIRT-1* activation pathways. Resveratrol and calorie restriction activate similar *SIRT-1*–mediated pathways whose actions result in prevention of common age-related diseases. (This figure has been taken from Markus and Morris, 2008) 9

The health benefits of resveratrol may derive from a group of substances called sirtuins, which activate or suppress members of the forkhead box O (FOXO) group of transcription factors (figure 1).

FOXOs activate or suppress specific genes leading to a decrease in apoptosis, an increase in anti-oxidative activities, DNA protection and anti-inflammatory effects [8,9,10,11,12,13]. Resveratrol modulates the expression of a number of genes involved in cell adhesion, muscle development, immune system regulation, neuroendocrine signalling, transcription and proteolysis [9,12,13].

Delayed onset muscle soreness (DOMS) is common and can occur independent of physical fitness levels. DOMS normally increases in intensity in the first 24 hours after exercise and peaks from 24 to 72 hours. Given the unknown aetiology of DOMS, many management modalities, including stretching, massage, cryotherapy or ibuprofen, have been proposed, but all have been found to be ineffective [15]. Resveratrol has several possible influences on tissues, including being an anti-oxidant and an anti-inflammatory, which could have an influence on DOMS (Figure 1).

No human studies have yet investigated the effects of resveratrol on immune response and DOMs. This pilot study therefore evaluated the effect of resveratrol on changes in immune response and DOMs after a marathon.

## METHODS

### Subjects and study design

This was a randomised double-blind placebo-controlled trial involving the recruitment of 8 well-trained distance runners participating in the 2010 London Marathon. Runners were recruited from regional and national running clubs.

Inclusion criteria were male gender and age 20 to 55 years. The male gender and age were chosen to ensure a homogenous cohort and to optimise safety of the use of resveratrol as a food supplement before a marathon run. Exclusion criteria included a past medical history of chronic inflammatory medical conditions, muscle disorders or heart conditions, and a drug history of immune suppresants or anti-inflammatories.

The study was undertaken with approval from the Ethics Committee of Queen Mary University of London. All subjects provided written informed consent before participation.

### Measurments and treatment allocation

#### Pre-marathon measurements

Before blood sampling a short questionnaire identified age, training volume and the exclusion criteria mentioned above. Intensity of training was evaluated by the average miles per week run three months before the London Marathon. Pre testing also included measurement of body weight and height to ensure that each participant had a similar dose per body weight of the Resveratrol.

Athletes were also asked to attend a blood taking session two days before the London marathon. WBC and CRP were processed within 8 hours of blood letting. Blood samples were taken from the antecubital fossa and were collected directly into Sartedt Monovette (EDTA KE; Serum Gel), and processed within the Division of Blood Sciences at Barts and The London NHS Trust. Measurements for delayed onset muscle soreness were assessment of current pain, using a visual analogue scale (VAS) pain score. Each subject was asked to evaluate his level of perceived muscle soreness, with ratings as follows: 0 cm indicating complete absence of pain, and 10 cm indicating extreme soreness, with noticeable pain and stiffness at all times.

After pre-testing, the eight participants were then randomly allocated to one of the two groups, as described below.

#### Blinding and Randomisation:

Before randomisation, two different sets of envelopes had been prepared by an independent person not associated with the study, containing 42 tablets for each participant. Four envelopes contained resveratrol tablets and Four envelopes contained placebo tablets. Each type of envelope was coded numerically (1 and 2), and the coding was not revealed to any of the study investigators until after data analysis. Another independent person then randomly allocated participants to blinded groups (labelled 1 or 2) using computer block randomisation, and participants received the appropriately labelled envelopes. Allocation concealment, as well as blinding of participants, assessor and data analyst were therefore assured during the entire study.

#### Treatment

Four subjects received a one week course of resveratrol 600 mg daily before the marathon, and four subjects received the same dose of placebo. Two 100 mg tablets were to be taken three times each day, with the last two capsules to be taken on the day of the race. Participants were asked not to alter their diet in any way, and were not given any nutrition or hydration advice prior to the race.

#### Post-marathon measurement:

Blood was taken within18–32 hours after the marathon to compare the inflammatory response. Blood was taken from the anti-cubital fossa in an upright sitting position. Analysis of blood samples was undertaken as for the pre-test. Measurements for delayed onset muscle soreness were also undertaken as for the pre-test.

#### Data Analysis

Frequency histograms of data in each group were inspected for normality, and were deemed non-normally distributed. A comparison of the change in each variable (post minus pre marathon value) across the two groups was performed, using the non parametric Mann-Whitey-U test.

### Results

#### Baseline characteristics

One participant was excluded from the study as he developed a viral infection one week before the marathon, and was treated with anti-inflammatory medications. This athlete had been allocated during to the Resveratrol group and was not included in the analysis. All seven participants included in the analysis were male and finished the marathon between 3 hours 10 minutes and 3 hours and 35 minutes.

No medical problems or injuries were recorded. The characteristics of the participants are shown in table 1. There was no significant age, weight or height difference between the groups, but there was a trend for a height difference. All participants had an average weekly training mileage of more than 30 miles in the 3 months before the marathon.

#### Safety Assessment

None of the seven participants reported any adverse effects during the study period or during the marathon itself.

#### Inflammation Assessment

##### White Blood Cell Count

**Figure 2:**
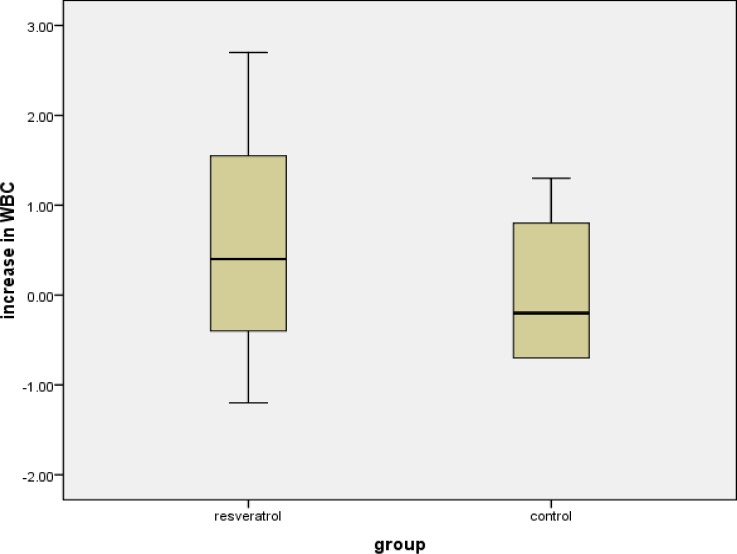
Box plot of the increase in WBC in resveratrol and control groups

There was no rise in the white blood cell count following the marathon in both groups combined. Comparing the increase of WBC between the resveratrol group and the placebo group there was no significant difference between the two groups. [p=0.857; median Resveratrol (IQR): 0.40 (−1.2 to 2.7); median Placebo (IQR): −0.2 (−0.7 to 1.05)] (Figure 2).

##### C Reactive Protein

**Figure 3:**
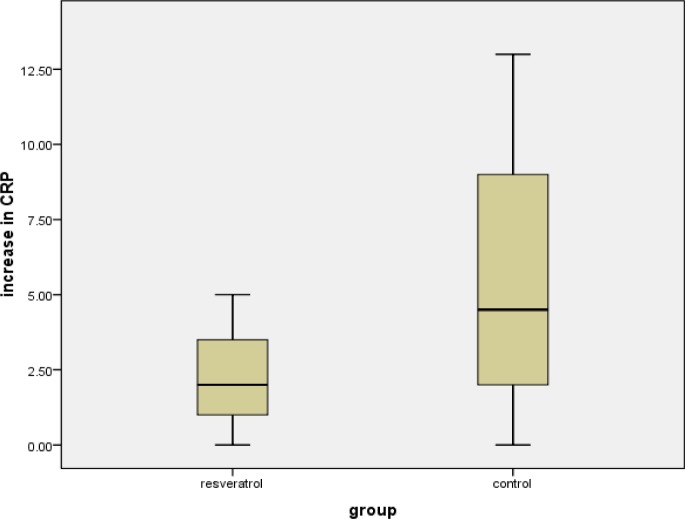
Box plot of the increase in CRP in resveratrol and control groups

There was a substantial increase in CRP in both groups combined, from before to after the marathon [median before (IQR): 5 (5 to 5); median after (IQR): 9 (5 to 10); p = 0.042]. Comparing the increase of CRP between the resveratrol group and placebo group showed no significant difference [median resveratrol increase (IQR): 2.0 (0.0 to 5.0); median placebo increase (IQR): 4.5 (1.0 to 11.0); p=0.629] (Figure 3).

#### Delayed Onset of Muscle Soreness

**Figure 4:**
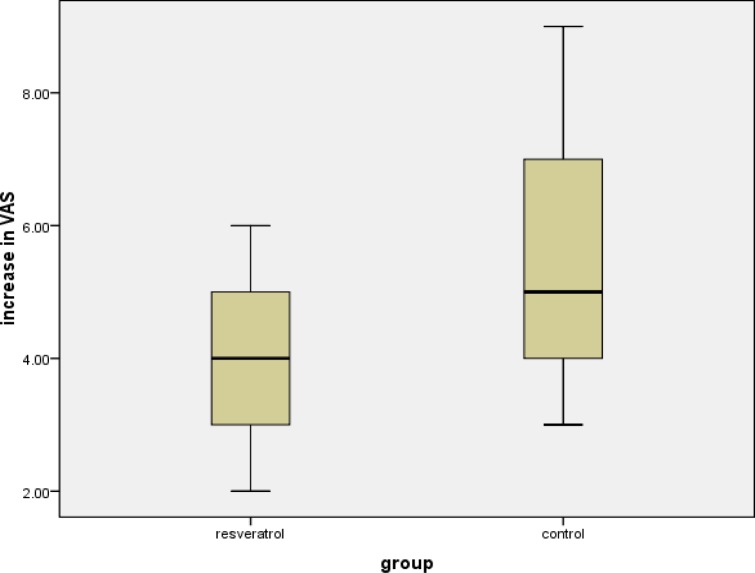
**Box plot of the increase in VAS Score in resveratrol and control groups**

Assessment of current muscle pain before and 24 hours after the marathon showed increased pain 24h after the marathon in both groups combined [median before 1 (1 to 2); median after 6.5 (3.75 to 8.5); p = 0.028]. There was no difference in the increase between the resveratrol group and the placebo group [median resveratrol increase (IQR): 4.0 (2.0 to 6.0); median placebo increase (IQR): 5.0 (3.0 to 9.0); p=0.70;] (figure 4).

## DISCUSSION

This study did not detect significant differences between placebo and resveratrol in terms of their effects on increases in WBC, CRP or delayed DOMS after the London Marathon.

There are several possible reasons for these results. First, a real difference could exist between resveratrol and placebo, but no difference was detected in our investigation. This could result from the low numbers of participants, and therefore a lack of statistical power. *Post hoc* power calculation showed that, to avoid a type II error, 17 runners in each group (34 in total) would be required for the CRP data. Further work with larger sample sizes is therefore required to confirm or refute this.

Secondly, the lack of detection of group differences could also be due to the timing of tests. Other studies have shown rises in WBC peaking 3 hours after the run, with a sustained increase over the next day up to 36 hours [2,3,4,7,21]. In the current study, blood was taken only once within 18–32 hours after the marathon, and so true effects may have missed.

Thirdly, the dose of resveratrol in the current study may have been insufficient to show effects [23]. Mayers [24] study fed mice a 0.1% resveratrol diet for 12 weeks, and found no physiological effects of resveratrol, whereas Murase [25] found physiological differences in mice with a 0.2% resveratrol diet for 12 weeks. Baur 8 found that long term treatment (over 1 year), despite a 10–20 fold lower daily dose than other studies [14], led to a remarkably improved insulin sensitivity and increased life span in mice fed a high fat diet. Therefore, it is possible that a one week course of resveratrol is not enough to influence the immune response after heavy exercise. In animal studies, a longer treatment time of 12 weeks prior the marathon appears to be a possible time frame for a follow up study. Future studies might also consider having two resveratrol groups with different daily doses.

A final reason for the inconclusive results could be that resveratrol has no influence on WBC, CRP or DOMs response to marathon running in humans. Animal studies have shown that resveratrol influences interleukins and prostaglandins [5, 11], and further human studies might include such parameters as potentially more sensitive markers.

A major strength of this study were the rigorous process of randomisation and blinding. Despite the small sample sizes, the groups were similar in terms of potential confounders such as training load and weight, and so threats to theinternal validity of the study were minimised.

As already highlighted, a limitation of this study was the low number of participants, which probably increased the risk of a type II error. Although higher numbers were planned, with several efforts to recruit participants, marathon runners seem to be extremely resistant to alterations in their diet or using any form of food supplements. This should be taken into account when planning further studies and more time should be spent in providing information about resveratrol.

## CONCLUSIONS

Despite basic scientific evidence for resveratrol as an anti-inflammatory food supplement, this pilot study could not detect a difference between resveratrol and placebo in terms of effects on post-marathon immune response or DOMS. Follow up studies should recruit at least 34 runners (17 in each group) to avoid a type II error, and consider a longer treatment time of resveratrol with a higher dose. The investigation of different serum indicators of inflammation, such as Interleukin-6, should also be considered.

## Figures and Tables

**Table1.** Characteristics of participants

	Resveratrol group (n=3)	Placebo group (n=4)	P value
Age (years)	49 (41–55)	54 (40–55)	0.857
Weight (kg)	70 (66–72)	70 (68–78)	0.857
Height (cm)	171 (153–177)	183 (182–185)	0.057
